# Single cell mapping of B and T cell dynamics in breast cancer lymph node metastasis

**DOI:** 10.3389/fbinf.2026.1833416

**Published:** 2026-08-12

**Authors:** Sajid Khan, Sabahat Jamil, Muhammad Hamza, Zarlish Attique, Suping Zhang

**Affiliations:** 1 Shenzhen Key Laboratory of Precision Medicine for Hematological Malignancies, Guangdong Key Laboratory for Genome Stability and Human Disease Prevention, Department of Pharmacology, School of Basic Medical Science, Base for International Science and Technology Cooperation, Carson Cancer Stem Cell Vaccines R&D Center, International Cancer Center, Shenzhen University Medical School, Shenzhen University, Shenzhen, China; 2 Department of Bioinformatics, Government Postgraduate College Mandian, Abbottabad, Pakistan

**Keywords:** B&T cross talk, breast cancer, chemokine-cytokine signaling, lymph node metastasis, ScRNA-seq

## Abstract

Metastatic breast cancer remains difficult to cure, and the way B and T lymphocytes adapt across metastatic niches especially under therapy remains insufficiently defined. Clarifying compartment specific immune remodeling may help explain resistance to PD-1/PD-L1 blockade and identify actionable targets. We performed an integrated meta-analysis of single cell RNA-seq datasets from normal breast tissue, primary tumors, tumor-draining lymph nodes (TLNs), and peripheral blood mononuclear cells (PBMCs), focusing on B and Tcell states. Immune composition differed notably by compartment. Tumors were enriched for effector CD8 states (CD8 cytotoxic 20.1%; CD8 activated 13.5%), whereas TLNs preserved larger naïve and memory reservoirs (CD4 naïve 40.7%; B naïve 11.4%; B memory 12.0%) and contained a higher B cell fraction than tumors (39.6% vs. 19.5%). Post therapy, PBMCs and TLNs showed increased BTLA-HVEM (TNFRSF14) checkpoint signaling and enhanced MIF-CD74 interactions with a shift from CD44 toward CXCR4, consistent with CXCR4 driven migratory and survival programs. In TLNs, TNFRSF14 signaling was unidirectional (B→T), absent in the reverse direction, and not detected in tumors. Clinically, higher tumor CXCR4 combined with lower TNFRSF14 was associated with shorter progression free survival in TCGA-BRCA, most evident in node positive, early stage disease. To target the BTLA-HVEM checkpoint axis, we performed structure guided *de novo* peptide design using the native HVEM (23-39) peptide as an active structural template, followed by docking and molecular dynamics simulations. The optimized De novo-P2 peptide showed stable and favorable interactions at the BTLA interface, supporting its potential as a competitive modulator of BTLA-HVEM signaling. These data define niche specific lymphocyte remodeling and implicate BTLA-HVEM and CXCL12-CXCR4 as candidate biomarkers and therapeutic targets linked to PD-1/PD-L1 resistance.

## Introduction

Breast cancer remains the most prevalent malignancy among women, distinguished by significant clinical and molecular heterogeneity. Lymph node metastasis represents a pivotal early event in disease progression, serving as a critical indicator of systemic dissemination and strongly correlating with reduced survival outcomes (Riggio et al., 2021; Xie et al., 2025). While localized breast cancer boasts a 5-year survival rate exceeding 99%, regional lymph node metastasis reduces this rate to approximately 87%, and distant metastasis further diminishes it to 32%, highlighting the prognostic importance of metastatic spread (American Cancer Society, Breast Cancer Survival Rates, August 2025) (Li et al., 2025). This metastatic transition not only signifies progression to advanced disease but also reflects complex interactions between tumor cells and their microenvironment, particularly immune cells, which play dual roles in either constraining or facilitating metastatic spread (Grant and Ferrer, 2025; Pena-Romero and Orenes-Pinero, 2022).

Lymph node metastasis in breast cancer involves a complex, coordinated cascade within the tumor microenvironment (TME). The tumor draining lymph node (TLN) microenvironment, a critical site of immune surveillance, undergoes significant remodeling during metastasis, fostering an immunosuppressive niche that supports tumor cell survival and dissemination (du Bois et al., 2021; Ginter et al., 2019). This dynamic interplay involves multiple immune cell types, including B and T lymphocytes, natural killer (NK) cells, macrophages, and dendritic cells, which engage in dynamic crosstalk with metastatic breast cancer cells. Understanding these cellular interactions within the immune TME is essential for elucidating the mechanisms driving tumor initiation and progression to lymph node metastasis (Ruocco et al., 2024; Alberts et al., 2021). While bulk genomic sequencing has identified pro and anti-inflammatory signatures, chemokines, and cytokines enriched in breast cancer metastases, these approaches fail to resolve the specific cellular sources of these signals or the interactions among immune subsets that shape the metastatic niche in advanced disease (Salemme et al., 2021).

Single cell RNA sequencing (scRNA-seq) enables detailed profiling of individual immune cells within normal breast tissue, primary tumors, and TLNs, revealing complex immune interactions in breast cancer and offering insights into reversing immunosuppression within metastatic lymph nodes to restore anti-tumor immunity (Mao et al., 2023). Previous studies have highlighted the dual roles of B and T cells in breast cancer: certain subsets, such as regulatory B cells (Bregs), secrete immunosuppressive cytokines that promote T-cell exhaustion and immune tolerance, while others drive anti-tumor immunity through antibody production and antigen presentation. Similarly, regulatory T cells (Tregs) in breast cancer may contribute to immune tolerance and poorer survival outcomes, whereas cytotoxic T cells with exhausted gene expression profiles often characterize an immunosuppressive TME (Xu et al., 2020). Lymph node resident stromal cells further amplify immunosuppression by producing chemokines (e.g., *CCL5*, *CXCL12*) that recruit pro-tumorigenic immune subsets and secrete cytokines such as *TGF-β* and *IL-10*, which suppress cytotoxic T cell activity and upregulate immune checkpoints like *PD-L1*. This immune editing process enables metastatic cells to evade immune detection, disrupt antigen presentation, and prime distant organs for colonization (Zou et al., 2023). Despite the central role of immune dysregulation in metastatic progression, comprehensive transcriptomic profiling comparing the primary and metastatic breast cancer TME at single cell resolution remains limited, with most studies focusing on unpaired samples or primarily analyzing malignant and stromal cells, leaving the evolution and interactions of B and T cell subsets across primary and metastatic sites underexplored (Lei et al., 2022; Liu K. et al., 2024).

In this study, we present a comprehensive single cell analysis of the immune landscape across primary breast tumors and TLN metastases using publicly available scRNA-seq datasets. By focusing on immune cells, we map the transcriptional heterogeneity and differentiation states of B and T lymphocytes across these compartments and disease states. Our integrative approach uncovers distinct immune cell compositions, signaling patterns, and ligand-receptor (LR) networks, emphasizing the compartment specific roles of *CXCR4* and *TNFRSF14* in immune suppression and T cell exhaustion. This work provides a detailed analysis of immune remodeling during metastatic progression in breast cancer and establishes a foundation for identifying context specific therapeutic targets beyond *PD-1*/*PD-L1* blockade. Building on the identification of BTLA-HVEM as an inhibitory immune checkpoint axis, we further used the resolved BTLA-HVEM interface to guide the design of BTLA binding peptides as candidate inhibitors aimed at disrupting HVEM mediated checkpoint signaling.

## Materials and methods

### Bulk transcriptomic data acquisition and preprocessing

To characterize the immunological context of breast cancer, bulk RNA sequencing data from the TCGA-BRCA cohort were analyzed, comprising 1,096 primary tumor samples (Xu et al., 2020). This dataset was used to assess the expression of cytokines, chemokines, and inflammatory markers associated with B and T cells. Additionally, curated pro-inflammatory and anti-inflammatory gene signatures were applied to evaluate their impact on tumor immune interactions and patient survival.

### Single cell RNA sequencing datasets

We curated and integrated single cell RNA-sequencing (scRNA-seq) datasets from four publicly available studies archived in the Gene Expression Omnibus (GEO) database (Supplementary Table S1). The dataset GSE161529 included 421,761 cells from 52 patients, with six lymph node metastases from estrogen receptor positive (ER^+^) tumors (Pal et al., 2021). Dataset GSE195861 profiled 13 breast cancer samples including six invasive ductal carcinoma (IDC) lymph node metastases and seven ductal carcinomas *in situ* (DCIS) samples (Tokura et al., 2022). GSE180286 comprised matched primary tumors and lymph node samples from five patients (Xu et al., 2021) and GSE263995 provided scRNA-seq profiles of 14 samples including pre and post chemotherapy treatment, across tumor infiltrating immune cells, tumor draining lymph nodes (TdLN), and peripheral blood mononuclear cells (PBMCs) (Jin et al., 2024). To assess whether our findings could be reproduced in an independent cohort, we analyzed the publicly available single-cell RNA-sequencing dataset GSE167036, which contains paired primary tumor and lymph node metastasis samples (n = 32) (Liu et al., 2022). These datasets enabled comparative analyses across tissue types and treatment stages.

### Single cell data processing and clustering

All scRNA-seq data were processed using the Seurat R package (v5.3.0) (Satija et al., 2015). Cells with fewer than 200 genes, fewer than 500 unique molecular identifiers (UMIs), or more than 20% mitochondrial content were excluded. Genes expressed in fewer than 5 cells were filtered out. Data normalization was performed using NormalizeData(), followed by scaling with ScaleData(). Highly variable genes (n = 2,000) were identified using FindVariableFeatures(). Dimensionality reduction was conducted via principal component analysis (PCA), selecting 30 principal components. Batch effects across datasets were corrected using Harmony (v1.2.3), and clustering was performed using FindNeighbors() and FindClusters() (Korsunsky et al., 2019). Dimensionality reduction and visualization were achieved using UMAP and t-SNE (RunUMAP() and RunTSNE()).

### Cell type annotation

Cell type annotation followed a two step approach, initial automated annotation using SingleR (v2.4.1) (Aran et al., 2019), followed by manual refinement with ScType, which utilizes marker gene lists for robust classification (Supplementary Table S2) (Ianevski et al., 2022). Epithelial cells were identified via *EPCAM* expression, and immune cells by *PTPRC* (*CD45*). Marker genes including *EPCAM, KRT7, KRT8, KRT18* (epithelial) and *CD3, CD4, CD8, CD14, CD19, CD68, FOXP3, NKG7*, and *GNLY* (immune) were used for cluster validation and classification. Cell type proportions were analyzed by condition-specific cell-type composition and statistical testing using the propeller framework (Speckle v1.6.0), with cell-type annotations, sample groups (Tumor, TLN, PBMC), and metadata columns (cell-type, sub-cell-type) as inputs for ANOVA based comparisons of differential cell-type proportions across conditions. Propeller framework uses variance moderated linear modeling for compositional data with Benjamini Hochberg FDR control.

### Immune cell subset identification and sub-clustering

To resolve immune cell heterogeneity, a second round of clustering was conducted on immune cells only. The Harmony corrected PCA space was used, with clustering resolutions ranging from 0.1 to 0.9. This analysis identified major immune populations: T cells (*CD3D, CD3E, CD3G, CD4, CD40LG, CD8A, CD8B*), B cells (*CD79A, CD79B, CD19, MS4A1*), plasmablasts (*JCHAIN, MZB1*), myeloid cells (*LYZ, CD68, FCGR3A, MARCO*), and natural killer (NK) cells (*NKG7, GNLY, TYROBP, PRF1*). T cells were further subclustered into naïve, memory, cytotoxic, activated, exhausted, regulatory (Treg), follicular helper (Tfh), central memory (Tcm), effector memory (Tem), and tissue resident memory (Trm) subsets. B cells were annotated as naïve, memory, plasma, and plasmablasts. Detailed marker gene lists are provided in Supplementary Table S2.

### Differential expression and functional annotation

Differential expression analysis was performed within each disease-state comparison to assess how gene expression varies across conditions. Cluster-specific differentially expressed genes were identified using Seurat’s FindAllMarkers() with DESeq2, MAST, and a two-sided unpaired Wilcoxon rank-sum test. To improve statistical robustness and limit multiple testing, pairwise contrasts (Normal vs. Tumor, Tumor vs. tumor-draining lymph node, and Normal vs. tumor-draining lymph node) were additionally evaluated using FindMarkers(). Genes were considered significant for a given cell type if they were detected in at least 10% of cells in either group (min.pct = 0.1), showed an absolute log2 fold change ≥0.25, and had a Bonferroni-adjusted p value ≤0.05.

### Functional enrichment analysis

To characterize disease state specific biological programs, we performed pathway and process enrichment. Over representation analysis (ORA) was conducted in clusterProfiler (v4.1.4) on differentially expressed gene sets (Yu et al., 2012). Gene ontology biological process enrichment was computed with enrichGO() using the human annotation database OrgDb = org.Hs.eg.db (loaded via library(org.Hs.eg.db)), with gene identifiers mapped as required. KEGG pathway enrichment was performed using enrichKEGG(), and WikiPathways enrichment was performed using enrichWP() for the human gene set collection. For all enrichment analyses, p values were adjusted using the Benjamini-Hochberg procedure, and terms were considered significant at FDR (adjusted p) < 0.05, requiring a minimum of ≥3 genes per term. In parallel, pathway activity at the cluster level was quantified using GSVA with Hallmark gene sets, using cluster averaged normalized expression to generate per cluster pathway activation scores.

### Gene regulatory network inference

Gene regulatory networks (GRNs) were constructed using pySCENIC (v0.12.1) (Van de Sande et al., 2020). Transcription factor target interactions were inferred via the GRNBoost2 algorithm. Motif enrichment analysis was performed using cisTarget and the Human Motif Database (v10). Regulons were filtered based on motif presence, and activity was quantified using area under curve (AUC) scores. Regulon specificity scores were calculated to identify differentially active transcriptional programs in B and T cell populations.

### Cell-cell communication analysis

Intercellular communication between B and T cells in tumor, TLN, and PBMC samples was inferred using CellChat (v2.1.2) (Jin et al., 2021). We focused on subtype-specific signaling changes, particularly differences in predicted communication between tumor and tumor-draining lymph node compartments. The analysis assessed ligand-receptor interactions across three signaling modalities: secreted signaling, extracellular matrix (ECM)-receptor interactions, and direct cell-cell contact. Following CellChat’s recommended workflow, each dataset was analyzed separately using default settings (significance threshold p = 0.05) and a minimum of 10 cells per group. Communication probabilities and interaction strengths were computed for each cell subset, and significant interactions (p < 0.05) were visualized using dot plots. Ligand-receptor expression patterns across compartments and cell types were displayed using ComplexHeatmap (v2.6.2) (Gu et al., 2016).

### Survival analysis

To evaluate the prognostic significance of immune gene expression, survival analysis was performed on the TCGA-BRCA cohort (n = 1,096). Gene signature scores were calculated using AUCell (v1.24.0), and survival curves were generated using the survival (v3.8.3) and survminer (v0.5.0) R packages for the optimal cutoff. Kaplan Meier plots were used to assess associations between gene signatures and overall survival outcomes in breast cancer patients.

### Peptide design and molecular dynamics simulations

The crystal structure of the BTLA-HVEM complex was obtained from the Protein Data Bank using (PDB ID: 2AW2; 2.8 Å resolution) (Compaan et al., 2005) ColabDesign/AFDesign was then used to design 17-residue peptides targeting the HVEM contacting surface of BTLA (Compaan et al., 2005; Anishchenko et al., 2021) Designs were prioritized by AlphaFold-Multimer confidence metrics (ipTM/pLDDT) and interface quality, and the top-ranked candidates were subjected to docking refinement using HADDOCK (HADDOCK score) and AutoDock Vina to evaluate binding orientation and interface contacts prior to molecular dynamics (MD) simulation (Jumper et al., 2021; Trott and Olson, 2009; van Zundert et al., 2016).

Three designed peptides (P1, YRDDDDCSSDYDDDCEP; P2, QQGRVKWRSIEYIKPEE; P3, KELEIYPRMRQLRKQQN) and a reference peptide corresponding to HVEM residues 23-39 (YRVKEACGELTGTVCEP) were complexed with BTLA, and each complex was prepared in CHARMM-GUI. Complexes were solvated in a dodecahedral TIP3P water box and neutralized with Na^+^ and Cl^−^ ions to a physiological concentration of 0.15 M. MD simulations were performed in GROMACS 2025.4 with the CHARMM36 force field. Systems were energy-minimized using the steepest-descent algorithm until the maximum force fell below 1,000 kJ·mol^−1^·nm^−1^, then equilibrated for 125 ps in the NVT ensemble following the CHARMM-GUI equilibration protocol, and simulated for 100 ns in the NPT ensemble. A 2 fs integration time step was used, with LINCS constraints applied to bonds involving hydrogen. Short-range van der Waals and Coulomb interactions were cut off at 1.2 nm, and long-range electrostatics were treated using the Particle Mesh Ewald (PME) method. The temperature was maintained at 303.15 K using the velocity-rescaling (V-rescale) thermostat, and production used isotropic C-rescale pressure coupling (τ_p = 5.0 ps, ref_p = 1.0 bar). Trajectories were analyzed for RMSD, RMSF, radius of gyration, solvent-accessible surface area, hydrogen bonding, and interface distance using GROMACS tools and in-house Python scripts, and structures were visualized in PyMOL and VMD (Hollingsworth and Dror, 2018).

## Results

### Metastatic breast cancer progression and immune remodeling

**FIGURE 1:**
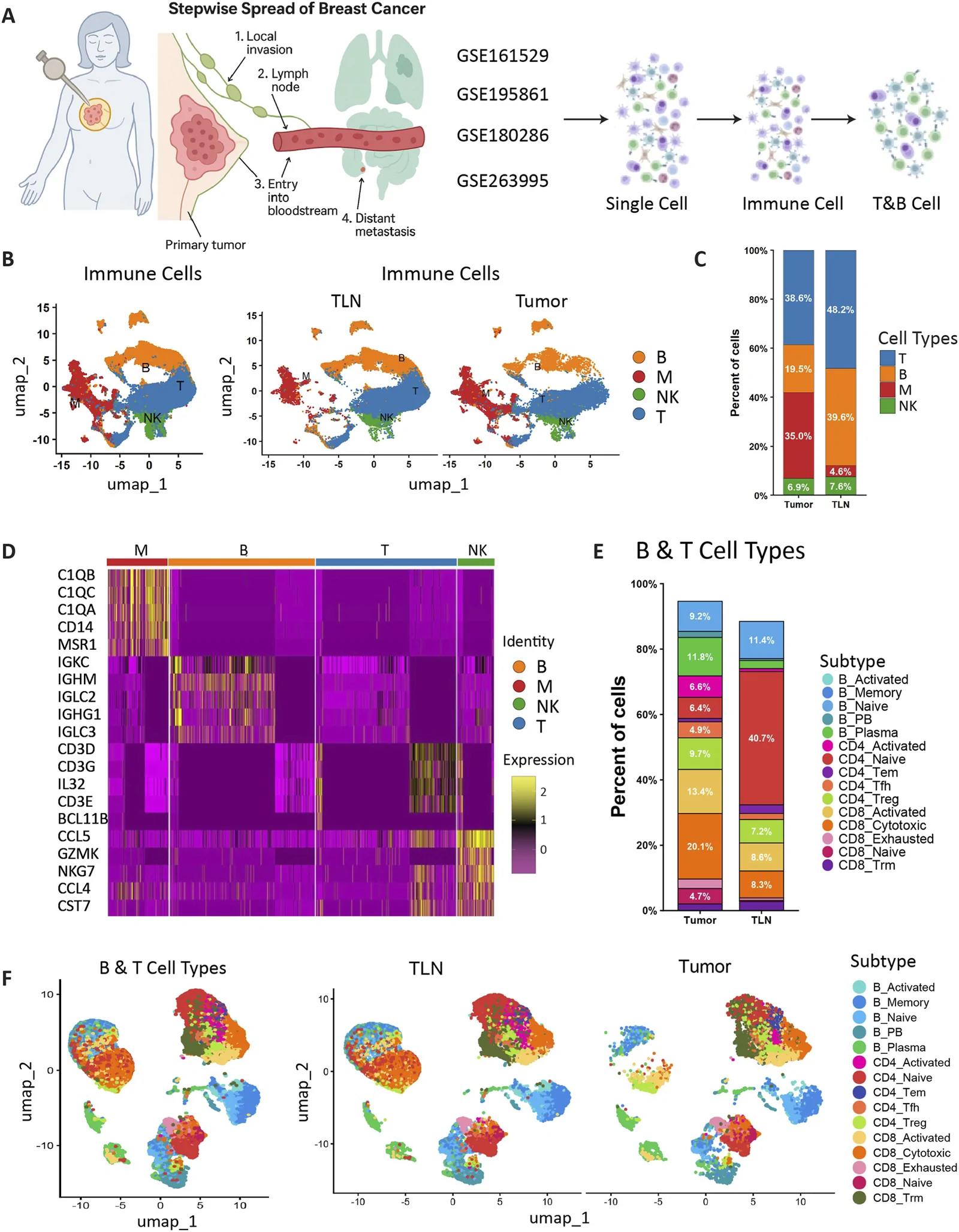
GEO cohort curation and single-cell immune (B&T) across tumor and tumor lymph node (TLN). **(A)** Study overview and dataset curation. Public scRNA-seq datasets were curated from GEO datasets. **(B)** UMAP of all immune cells annotated by major lineages (B, T, NK, M→myeloid cells). Right, split UMAPs by condition (Tumor vs. TLN) highlight compartmental shifts. **(C)** Stacked bar charts quantify lineage and immune-subtype proportions across conditions, illustrating redistribution of subsets between Tumor and TLN. **(D)** Heatmap of top differentially expressed genes per immune lineage and B/T-cell sub-cluster, showing scaled expression of immune clusters markers. **(E)** Stacked bar charts quantify lineage and B/T-subtype proportions across conditions, illustrating redistribution of subtypes between Tumor and TLN. **(F)** UMAPs focused on lymphocyte populations. Left, combined B and T cells; middle and right, compartment resolved maps (TLN and Tumor) reveal distinct B cell (naïve, activated, plasmablast/plasma) and T cell (naïve, effector memory, cytotoxic, exhausted, Tfh, Treg) distributions.

We analyzed four publicly available single cell RNA sequencing datasets from GEO that include primary tumors and lymph node metastases from breast cancer patients (Figure 1A, Materials and Methods). After quality control, batch correction, and principal component analysis, immune cells from tumor and tumor draining lymph node were visualized with UMAP (Figure 1B). In tumors, immune cells comprised 38.6% T cells, 6.9% natural killer cells, 35.0% myeloid cells, and 19.5% B cells (Figure 1C). In tumor draining lymph node, the composition was 48.2% T cells, 39.6% B cells, 7.6 percent natural killer cells, and 4.6% myeloid cells (Figure 1C). B cell proportions were significantly higher in tumor draining lymph node than in tumors, and T cell proportions were also higher in tumor draining lymph node with a smaller but significant difference (Supplementary Table S3).

For downstream analyses we focused on B and T cells, retaining 59,101 high quality B cells and 83,884 T cells from the primary dataset. For validation, cells annotated as B cells and T cells in the independent GSE167036 dataset were analyzed, comprising 24,998 B cells and 54,736 T cells, respectively. In addition, the treatment validation dataset GSE263995 included 23,740 B cells and 43,665 T cells (Supplementary Table S1) (Jin et al., 2024). A heatmap shows marker gene patterns used to define major immune lineages and supports precise annotation within the microenvironment (Figure 1D). Clusters were annotated with ScType using canonical markers (Supplementary Table S2). B cells were resolved into B Naive, B Memory, B Plasma, B Activated, and B PB. T cells were resolved into CD4 Naive, CD4 Tcm, CD4 Tem, CD4 Treg, CD4 Tfh, CD8 Activated, CD8 Cytotoxic, CD8 Exhausted, and CD8Tcm (Figure 1D).

Cell type proportion analyses showed clear differences between tumor and tumor draining lymph node. Plasma B cells at 11.8 %, CD8 Activated at 13.5 %, and CD8 Cytotoxic at 20.1 % were enriched in tumors, whereas B Naive at 11.4 %, B Memory at 12.0 %, and CD4 Naive at 40.7 % were enriched in tumor draining lymph node (Figures 1E,F). These findings are consistent with reports of B cell enrichment in triple negative, HER2 positive, and luminal like breast cancer relative to normal tissue, and indicate even greater B cell dominance in tumor draining lymph node compared with tumors (Sun et al., 2025). T cell analyses showed higher proportions of CD4 Treg, CD8 Cytotoxic, and CD8 Exhausted in tumors, while tumor draining lymph node contained more CD4 Naive, CD4 Tfh, and CD8 Activated, consistent with immune priming in the lymphatic niche (Supplementary Figure S1). Cell type proportion analysis of the GSE167036 dataset showed consistent differences between tumor and lymph node compartments. Lymph nodes contained higher proportions of B cells and T cells, accounting for 29.1% and 54.9% of cells, respectively, whereas tumors showed lower proportions of B cells and T cells, comprising 9.5% and 33.5%, respectively. These findings confirm compartment specific differences in immune cell composition (Supplementary Figure S2).

### B and T cell remodeling across tumor and lymphoid tissues

**FIGURE 2:**
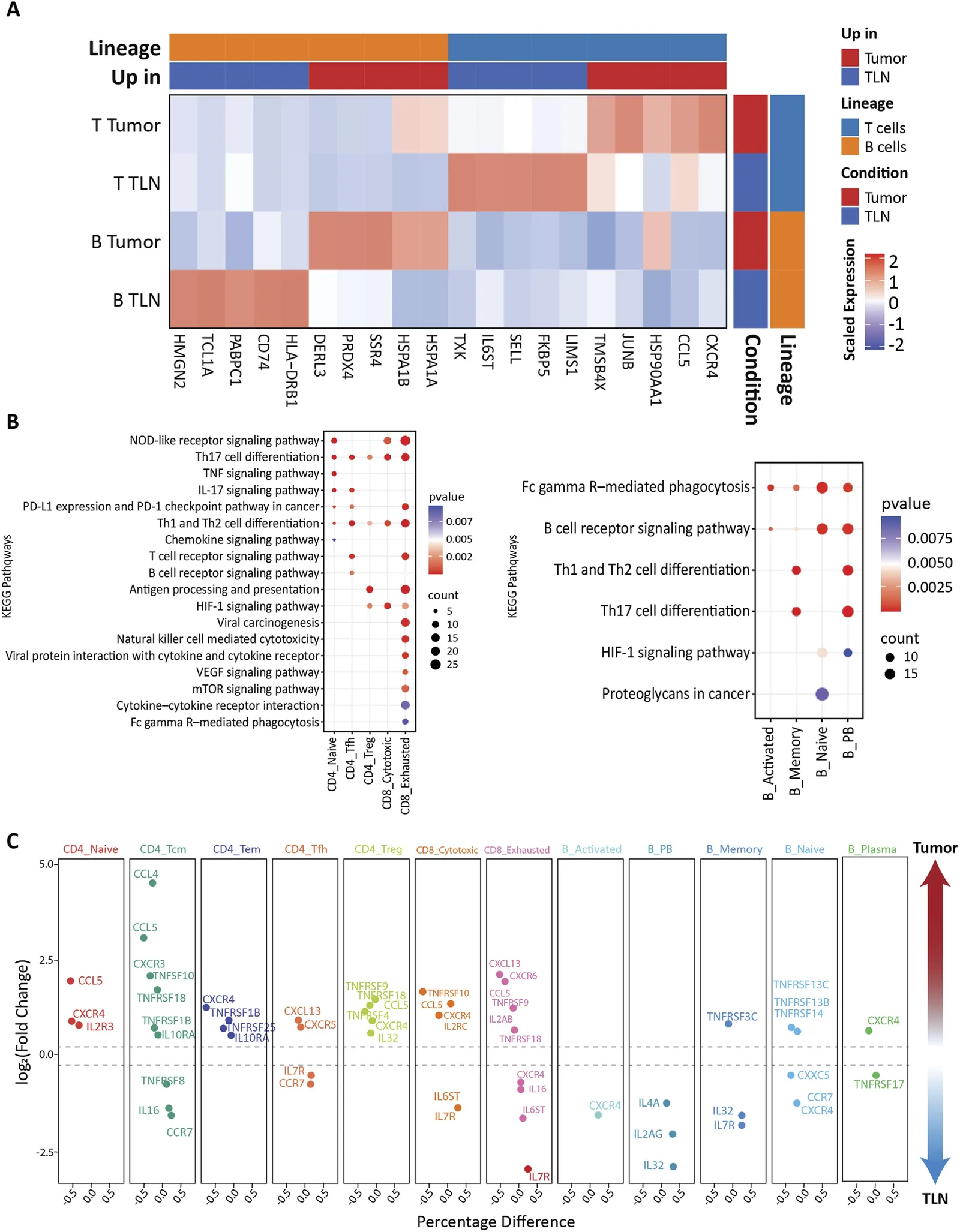
Chemokine landscape and pathway enrichment across B and T cell subpopulations. **(A)** Heatmap of scaled expression for shared and condition specific genes in B and T cells, comparing tumor and tumor lymph node (TLN) compartments. **(B)** KEGG pathway enrichment across T cell (left) and B cell (right) subpopulations. Enriched immune and stress response programs include TCR/BCR signaling, cytokine inflammatory pathways (TNF/IL-17), chemokine checkpoint signaling, and HIF-1/VEGF/mTOR associated responses, dot size indicates gene count and color indicates p value. **(C)** Expression patterns of chemokine and cytokine families (CXCL, CCL, IL, IFN) across B and T cell subsets and compartments, revealing subtype and tissue specific gradients consistent with differential recruitment.

Differential gene expression identified 144 genes upregulated in tumors and 83 in tumor draining lymph node using log2 fold change greater than 1 and adjusted P less than 0.05. T cells exhibited more pronounced changes than B cells. The heatmap highlights discrete patterns separating tumor cells from lymph node derived cells. In tumor infiltrating T cells, we observed upregulation of *CXCR4, CCL5, IL6ST, HSP90, JUNB*, and *TMSB4X*. In tumor draining lymph node T cells, *LIMS1, FKBP5, SELL, IL6ST,* and *TXK* were increased. In tumor associated B cells, secretory pathway and protein quality control genes *HSPA1A, HSPA1B, SSR4,* and *DERL3* were elevated, whereas lymph node B cells were enriched for antigen presentation and identity genes *HLA DRB1, CD74, PABPC1, TCL1A,* and *HMGN2*. These patterns indicate enhanced chemokine driven trafficking and cytokine responsiveness in T cells, and active antigen presentation and secretory adaptation in B cells within the tumor microenvironment (Figure 2A) (Sun et al., 2025; Delclaux et al., 2024).

### Immunotherapy induced changes

We assessed differential expression in B and T cells before and after immunotherapy. In pre-treatment B cells, antigen presentation genes *HLA DRB5, IGHG3*, and *IGLC1* or *IGLC2* and *TGF* beta signaling genes *ID2* and *SPARC* with fold change 2.10 and 3.53 were highly expressed. Transcription factors *ID2, FOSB,* and *NFAT5* were upregulated, suggesting activation of JAK STAT signaling and immunosuppressive B and T cell differentiation pathways (Mahjoor et al., 2023). Post-treatment B cells showed elevated interferon response genes *XAF1, IFITM1, MX1,* and *TRIM22*. In T cells, pre-treatment subsets expressed *HLA DRB5, HLA DRA, ERAP2,* and cytotoxic effectors *GZMK, GZMH, SYTL3,* and *CCL4*. Post-treatment T cells upregulated TCR signaling components *LAT, TRAF3IP3, CD48,* and *PTPN6* and interferon response genes *MX1, IFITM1 or IFITM2,* and *RNASET2*. Secretome profiling showed immunomodulatory signatures in B cells that included *IL7R, IL32, IL2RG, CXCR4, CCR7,* and *TGFB1,* and in T cells that included *CCL2, CCL4, CCL5, CCL8, CXCR3 or CXCR4, CXCL13, IL32,* and *IL2RG* (Supplementary Excel File) (Sun et al., 2025; Delclaux et al., 2024).

Pathway enrichment analysis revealed dual roles for NOD like receptor signaling in tumor progression and antitumor immunity. T cell pathways included Th1, Th2, and Th17 differentiation, with Th2 and Th17 linked to pro tumor roles and Th1 to antitumor activity. Subcluster analyses showed CD8 exhausted T cells enriched in TCR, cytokine receptor, chemokine, and VEGF or mTOR signaling, CD8 cytotoxic T cells with HIF1 activation, and CD4 naive and T follicular helper cells with TNF or IL17 pathway enrichment predominantly in lymph node (Figure 2B). In B cells, Fc gamma receptor mediated phagocytosis and BCR signaling were enriched in naive and plasmablast populations, with plasmablasts also engaging HIF1. Tumor samples showed increased PD1 blockade and costimulatory signaling, whereas lymph node samples were enriched for VEGFA or VEGFR2, TNF alpha, and TGF beta pathways. Both compartments exhibited type II interferon and interleukin signaling (Figure 2B).

Gene set enrichment analysis of pre versus post-treatment differentially expressed genes showed enrichment in T cells for PD1 blockade, TCR and costimulatory, type II interferon, and *IL2, IL3, IL4, IL6, IL7,* and *IL9* pathways. In B cells*, VEGFA* or *VEGFR2, BCR, TGF* alpha or beta, type II interferon, and *IL1, IL3, IL4, IL7, IL9,* and *IL26* pathways were upregulated, with negative enrichment for *TNF* and *IL6* regulatory pathways in both lineages. Gene ontology highlighted immune regulation, lymphocyte activation, and B or T cell signaling (Supplementary Figures S3A, B) (Patysheva et al., 2025).

Cytokine and chemokine profiles differed between tumor and lymphoid compartments. In tumors, CCL3, CCL4, and *CCL5* were highly expressed in CD4 Tcm, CD4 Naive, CD4 Treg, and CD8 Exhausted subsets. *CXCR4* and *CCR7* were prominent in both compartments, with *CXCR4* upregulated in CD4 Tem, CD4 Treg, CD8 Exhausted, B Activated, and B Plasma subsets, validated in TCGA BRCA with log2 fold change 1.134 and adjusted P 2.78 × 10^−28^ (Figure 2C). CXCR3, linked to effector T cell recruitment, was enriched in CD4 Tcm, CD8 Cytotoxic, and CD8 Exhausted. *TNFRSF14* was consistently downregulated in tumors and lymph node, particularly in B Naive cells with TCGA BRCA log2 fold change minus 1.35 and adjusted P 4.53 × 10^−40^. These signatures suggest a tumor environment skewed toward immune evasion, whereas lymph nodes maintain a more balanced, activating profile.

### Lymph node immune crosstalk is defined by enhanced lymphocyte signaling networks

**FIGURE 3:**
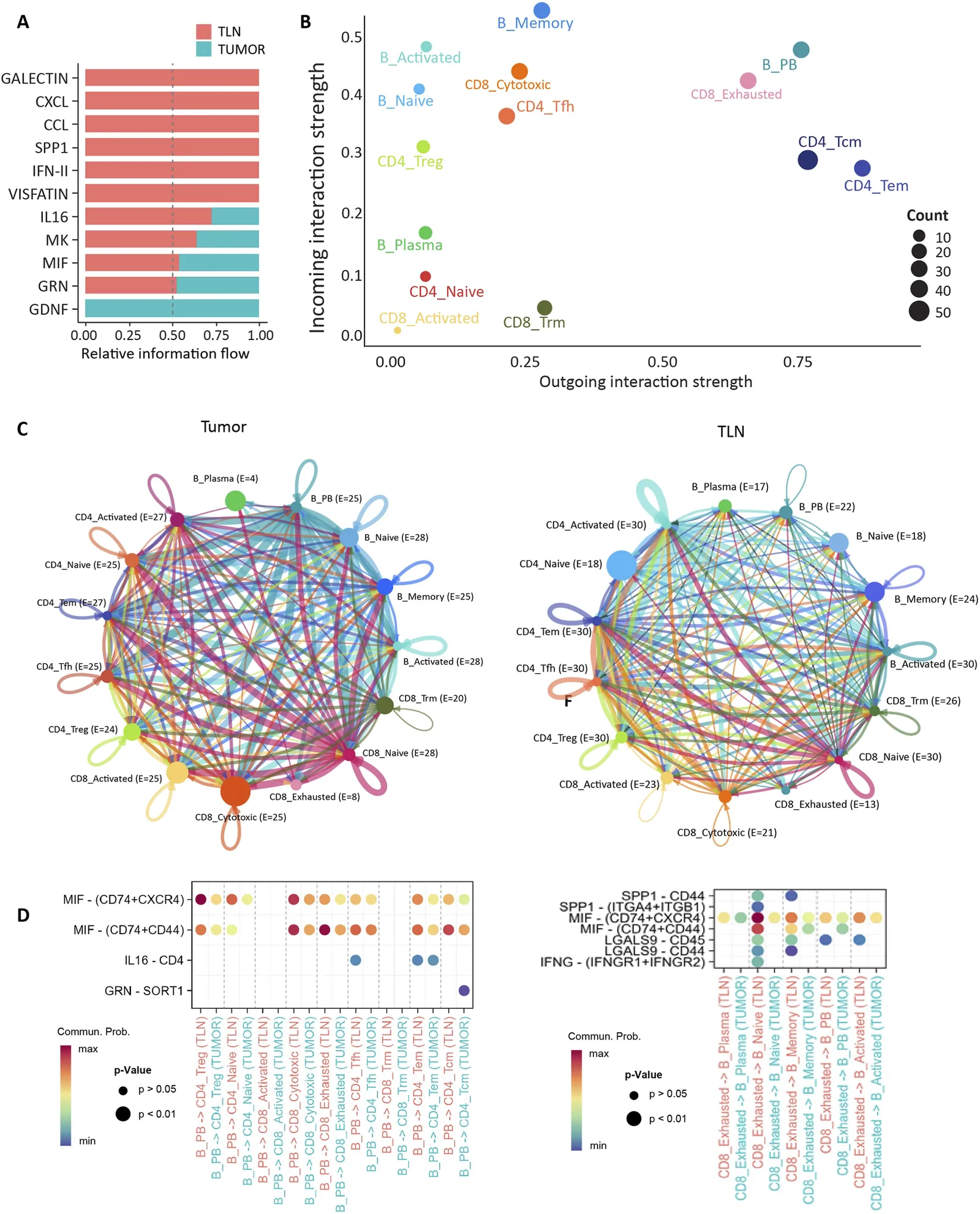
Cell cell communication networks across tumor and TLN microenvironments. **(A)** Pathway level information flow comparison between tumor and TLN. Bar plot shows integrated signal intensity per pathway; red indicates TLN enriched pathways and blue indicates Tumor enriched pathways (proportion and absolute contribution are reported). **(B)** Sender receiver role map for lymphocyte subtypes. Each point represents a cell subtype positioned by its “incoming” versus “outgoing” interaction strength; point size indicates the number of significant LR pairs for that subtype. **(C)** Circos map of B&T cell interactions inferred with CellChat, shown separately for Tumor and TLN compartments. Edge count (E = n) denotes the total number of ligand receptor (LR) interactions. Colors indicate the cell types involved, and line thickness represents the communication strength. **(D)** LR probability matrix depicting pairwise communication probabilities across B&T cell subtypes. Dot size encodes statistical significance (larger = p < 0.01), and dot color reflects communication probability/average LR expression (see scale).

We analyzed cell to cell communication to characterize immune crosstalk in tumor draining lymph nodes during breast cancer immunotherapy. Chemokine families *CXCL* and *CCL*, osteopontin *SPP1*, and type II interferon dominated in lymph node compared to tumor, indicating that lymph node act as hubs of immune activity (Figure 3A). CD4 Tfh, CD4 Treg, CD4 Naive, CD8 Cytotoxic, CD8 Trm, B Memory, B Activated, and B Naive showed the highest incoming signal intensities. B Plasma and CD8 Exhausted acted as both major senders and receivers (Figure 3B). Signaling networks were stronger in lymph node than in tumors (Figure 3C). LR pairing revealed that B Naive and CD4 Naive primarily responded to MIF and MK, that CD4 Tcm and CD4 Tfh responded to *IL16*, *GDNF*, and *VISFATIN*, and that CD8 Cytotoxic and CD8 Exhausted responded to CXCL ligands. Outgoing signals identified CD4 Tcm, CD4 Tem, CD4 Tfh, CD8 Cytotoxic, and B Memory as primary ligand sources that secreted *MK, IL16, CXCL, SPP1, GRN*, and *VISFATIN* (Supplementary Figure S4).

LR mapping identified a prominent *MIF*−*CD74* co-receptor signaling axis in the lymph-node compartment, particularly through *MIF*−*(CD74*−*CXCR4*) and *MIF*−*(CD74* *−* *CD44).* At the subset level, strong interactions were observed between CD8 cytotoxic T cells and B/plasma cells, together with directional signaling from CD8 exhausted T cells to naive B cells, confirming that T cell derived *MIF* signaling may contribute to B cell regulation within the lymph node microenvironment (Figure 3D). This pattern was supported by the independent data analysis of GSE167036 for higher predicted communication probability of *MIF-(CD74-CXCR4)* in T cell to B cell signaling. Overall, LR based B cell and T cell global communication analysis indicated stronger and more coordinated cell-cell signaling in the lymph node than in the tumor (Supplementary Figure S5).

These findings also align with recent single cell and proteomic studies that show enhanced *MIF-CD74-CXCR4* communication in lymph node after immunotherapy and with preclinical evidence that disrupting the *MIF CD74* axis can reprogram lymph node immune states to enhance antitumor immunity (Liu L. et al., 2024; Pellegrino et al., 2024).

### Chemotherapy drives immune remodeling and alters lineage composition across the tumor lymph node axis

**FIGURE 4:**
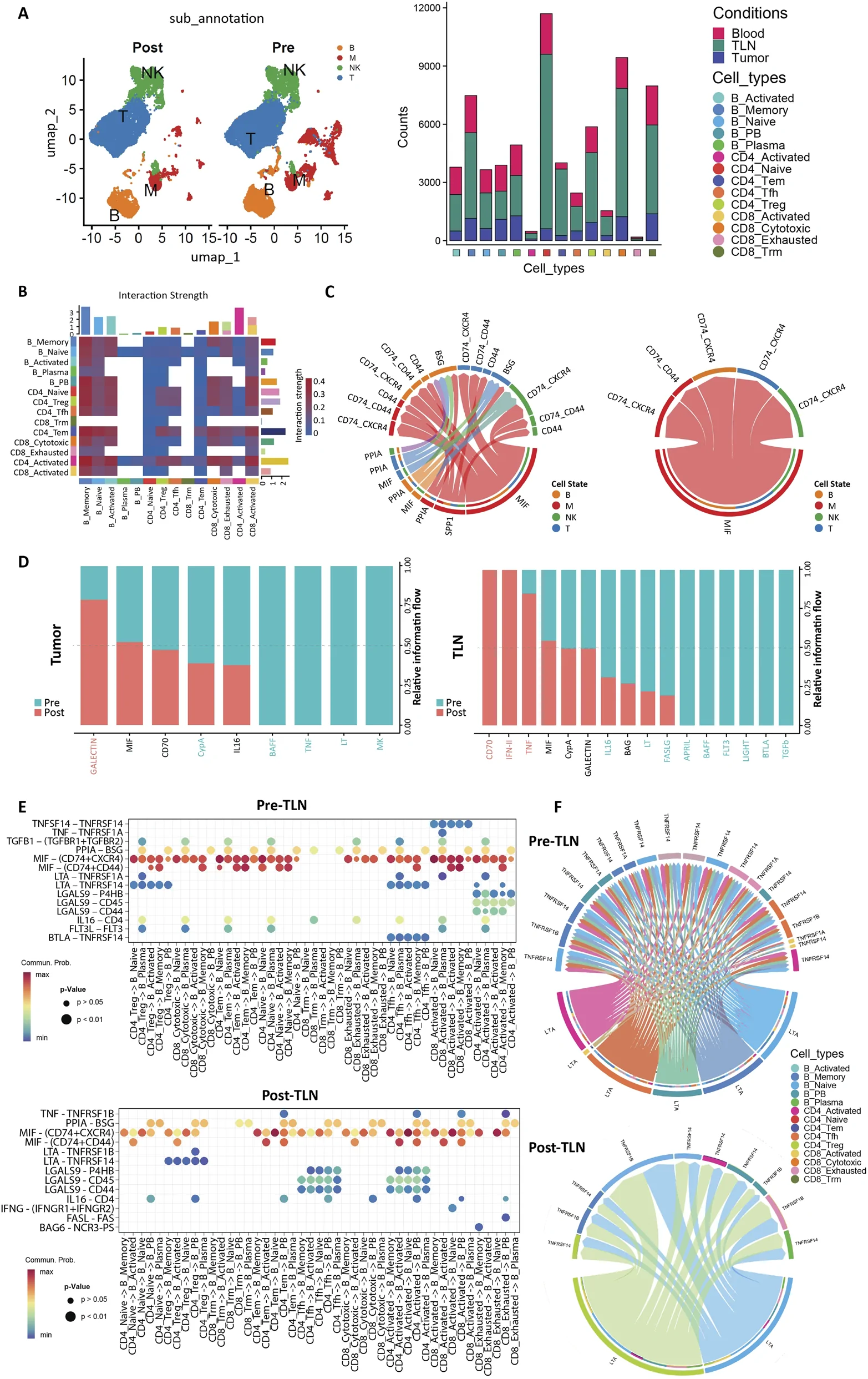
Test cohort (GSE263995): therapy responsive immune composition and communication across tumor, TLN. **(A)** UMAP of all immune cells, colored by major lineages (B, T, NK, M) and split by Pre vs. Post treatment (left). Stacked bar plot (right) quantifies lineage and lymphocyte-subset proportions across Tumor, TLN, and PBMC compartments for each treatment state. **(B)** Directed interaction matrices. Left: subtype level heat map of communication strength (rows = senders/outgoing; columns = receivers/incoming); marginal bars summarize row/column totals. **(C)** Chord (Circos) diagrams summarizing aggregate B-T cell communication. Right: Tumor networks are dominated by MIF centered signaling (via CD74-CXCR4/CD44) together with PPIA-BSG/CD147 interactions; Left: TLN networks are primarily MIF driven. **(D)** Signaling pathway enrichment (relative information flow) comparing Tumor (left) and TLN (right) across treatment states, with blue indicating Pre-therapy and red indicating Post-therapy. Bars summarize compartment-level shifts in signaling programs (e.g., MIF, CD70, INF-II, TNF). **(E)** Dot plots of TLN L-R interactions Pre-therapy (top) and Post-therapy (bottom) inferred with CellChat. Each dot represents an L-R pair between sender and receiver subsets; color encodes communication probability (scaled, blue = low, red = high), and dot size encodes statistical significance (larger = smaller p-value; e.g., p < 0.01). Representative families include MIF-CD74-CXCR4/CD44, BTLA-TNFRSF14 (HVEM), LTA-TNFRSF14, and CXCL/CCL chemokine axes. **(F)** Chord (Circos) maps of LTA-TNFRSF14 (LTα-HVEM) ligand-receptor signaling in TLN Pre-therapy (left) and Post therapy (right), summarizing directional interactions among B-T cell subsets.

Using GSE263995, we examined chemotherapy associated remodeling of lymphocyte composition and signaling. Overall architecture remained stable, yet B Memory, CD4 Naive, CD4 Tem, CD4 Treg, CD8 Cytotoxic, and CD8 Trm expanded significantly in lymph node after chemotherapy (Figure 4A). Pretreatment samples were enriched for inflammation, immune evasion, and metastasis signatures, whereas post-treatment profiles favored proliferative control and growth suppressive programs, particularly in CD8 Cytotoxic and B Plasma cells (Supplementary Figure S6). Tissue level communication mapping identified lymph node as highly interactive hubs, with reduced signaling in tumors. B cell subsets B Memory, B Activated, and B Naive showed the strongest incoming communication, while CD4 Activated and CD4 Tem were dominant signal senders (Figure 4B).

The *MIF-CD74-CXCR4* or *CD44* complex was primarily expressed by macrophages in both tumors and lymph node. In tumors it was also detected in B and T cells together with the *PPIA BSG* interaction. In lymph node, *MIF* complex expression was limited to macrophages. *IL16* was selectively downregulated in tumors after treatment. Pretreatment tumors expressed enriched MK and *LT*, and lymph node showed stronger *TGF beta*, *BTLA*, and *LIGHT* signaling (Figures 4C,D). At the subtype level, B and T cells in lymph node engaged in *MIF* mediated signaling before treatment, which decreased after treatment (Figure 4E). Persistent interactions in lymph node involved *MIF, PPIA,* and *LGALS9*, accompanied by a compositional shift toward B Plasmablast and CD4 Tem. After treatment there was a directional loss of *TNFRSF14* signaling. B to T cell communication via *TNFRSF14* persisted in lymph node, T to B signaling was lost, and tumors lacked *TNFRSF14* signaling in both directions. This selective disruption, given the role of *TNFRSF14* in T and B cell co-stimulation, suggests chemotherapy induced immunosuppression and aligns with prior single cell studies that reported expansion of memory like and cytotoxic lymphocytes in lymph node and rewiring of *TNF* receptor signaling including *HVEM* or *TNFRSF14*. Preclinical evidence supports the requirement for *TNFRSF14* in effective T and B cell activation, with impairment linked to immune dysfunction after treatment (Supplementary Figure S7) (Fransen et al., 2018; Yoo et al., 2022). Pretreatment, the *LTA TNFRSF14* complex was broadly active in lymph node among B Naive, B Plasmablast, and CD4 subsets including CD4 Treg, CD4 Tfh, and CD4 Activated. After chemotherapy this interaction was restricted to CD4 Treg and B Plasmablast, indicating reduced *TNFRSF14* mediated communication (Figure 4F). These data show that chemotherapy reshapes composition and rewires cell communication across the tumor to lymph node axis.

### *CXCR4* and *TNFRSF14* expression patterns serve as predictive markers of disease progression and immune suppression

**FIGURE 5:**
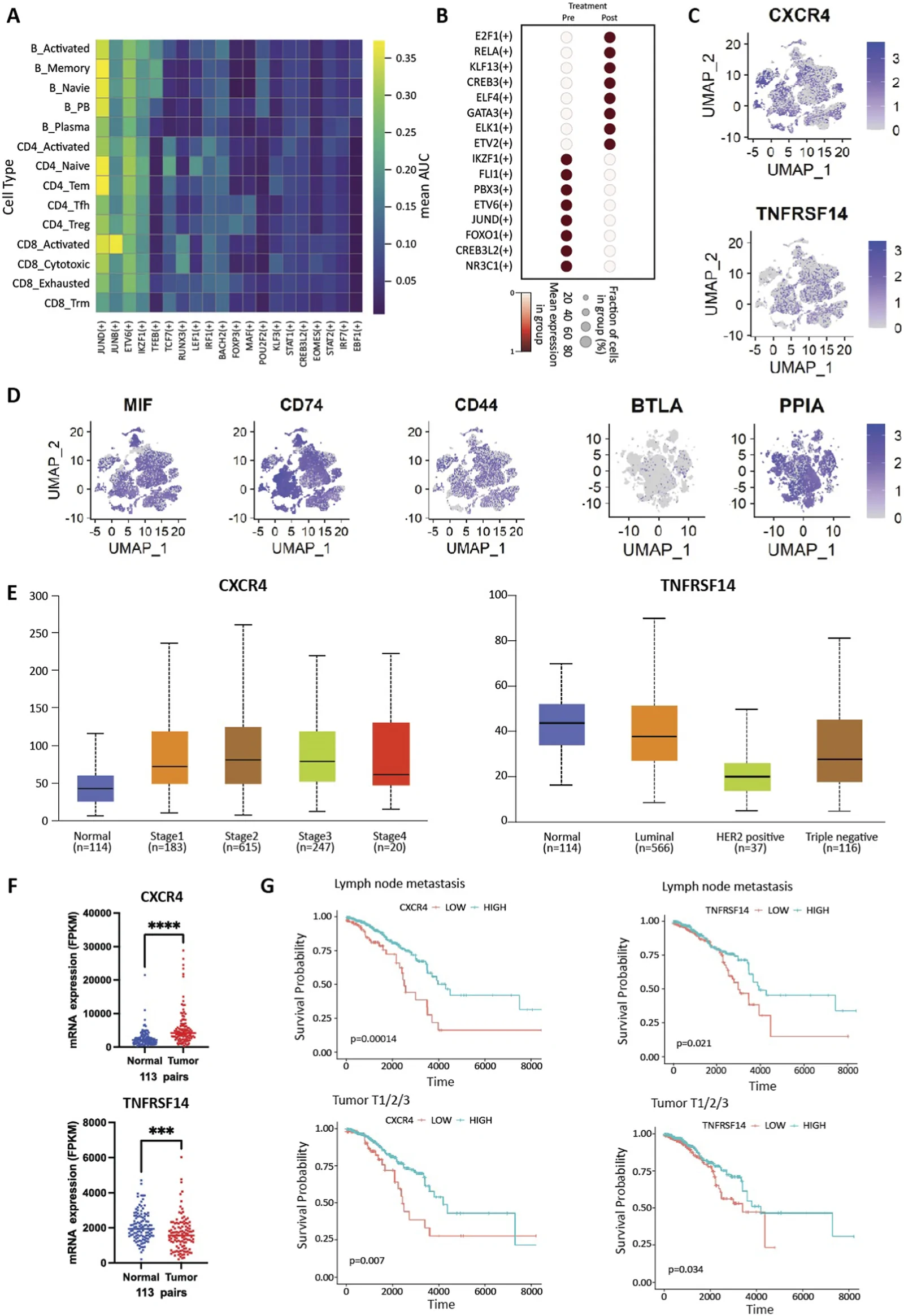
Cell type resolved immune programs in tumor and TLN, highlighting CXCR4 and TNFRSF14 expression and prognostic impact. **(A)** Heatmap of regulon activity scores (mean AUC) across immune subsets, highlighting transcription factor programs enriched in specific states (e.g., JUN, FOS, RELA, KLF13) in B and T cells. **(B)** Dot plot of differentially active regulons pre vs. post-treatment. Dot size indicates the fraction of cells with an active regulon; color encodes significance (log-adjusted *p* value or enrichment score, as indicated. **(C)** UMAP feature plots showing spatial expression of CXCR4 and TNFRSF14 (HVEM), broadly distributed and enriched within tumor infiltrating B and T cells. **(D)** UMAPs for MIF, CD74, CD44, BTLA, and PPIA, confirming compartment and subset-specific expression of checkpoint and ligand components implicated within specific immune cell signaling clusters. **(E)** Boxplots showing CXCR4 expression across clinical stages I-IV and TNFRSF14 across molecular subtypes (Luminal A/B, HER2^+^, TNBC), highlighting differential expression relative to normal breast tissue. Boxes depict median and interquartile range; significance by Wilcoxon rank-sum test. **(F)** Paired tumor normal comparison (FPKM) in 113 matched samples demonstrates significantly higher mRNA levels of CXCR4 (****p < 0.0001) and TNFRSF14 (***p < 0.001) in tumors. **(G)** Kaplan-Meier analyses stratified by high vs. low (median split) expression of CXCR4 (left) and TNFRSF14 (right). Higher expression associates with poorer survival outcomes, shown across strata of lymph-node metastasis and tumor stage (T1–T3).

We evaluated *CXCR4* and *TNFRSF14* as predictive biomarkers by analyzing transcriptional networks and expression across lymphocyte subsets. *JUND* was strongly active in B Memory, B Naive, B Plasmablast, CD4 Naive, and CD4 Tem. *JUNB* and *JUND* were co enriched in CD8 Activated. *ETV6* showed moderate but widespread influence across most subsets, indicating a role in lymphoid differentiation (Figures 5A,B). Before chemotherapy, regulatory networks were dominated by transcription factors that support immune homeostasis and memory survival, including *NR3C1, CREB3L2, FOXO1,* and *IKZF1*, and by activation threshold regulators including *JUND, ETV6, PBX3,* and *FLI1*. After chemotherapy, the landscape shifted toward proliferative and effector regulators including *E2F1, RELA, KLF13, CREB3, ELF4, GATA3,* and *ELK1,* consistent with enhanced T cell activation and lineage differentiation (Yoo et al., 2022).

UMAP visualization showed widespread expression of *MIF, CD74,* and *CD44* across B and T cell clusters, and broad expression of *PPIA*, with cell type specific patterns for *BTLA* and *TNFRSF14* (Figures 5C,D). *CXCR4* was notably elevated in breast cancer, with tumor cells showing nearly twice the levels of normal tissue. *CXCR4* peaked in stages I to III and declined in stage IV. *TNFRSF14* was higher in normal than tumor, with the lowest levels in HER2 positive and triple negative subtypes (Figure 5E). In 113 TCGA *BRCA* patients, tumors showed *CXCR4* upregulation with P less than 0.0001 and *TNFRSF14* downregulation with P equal to 0.0006 (Figure 5F). In 1,096 TCGA BRCA patients, high *CXCR4* or *TNFRSF14* expression correlated with reduced progression free survival, particularly in patients with lymph node metastases and in stages I to III (Figure 5G). These data highlight a clinically significant pattern in which high *CXCR4* expression drives progression and metastasis, while *TNFRSF14* loss suggests weakened immune stimulation, positioning both as prognostic biomarkers and therapeutic targets.

### Molecular dynamics evaluation of designed peptides

**FIGURE 6:**
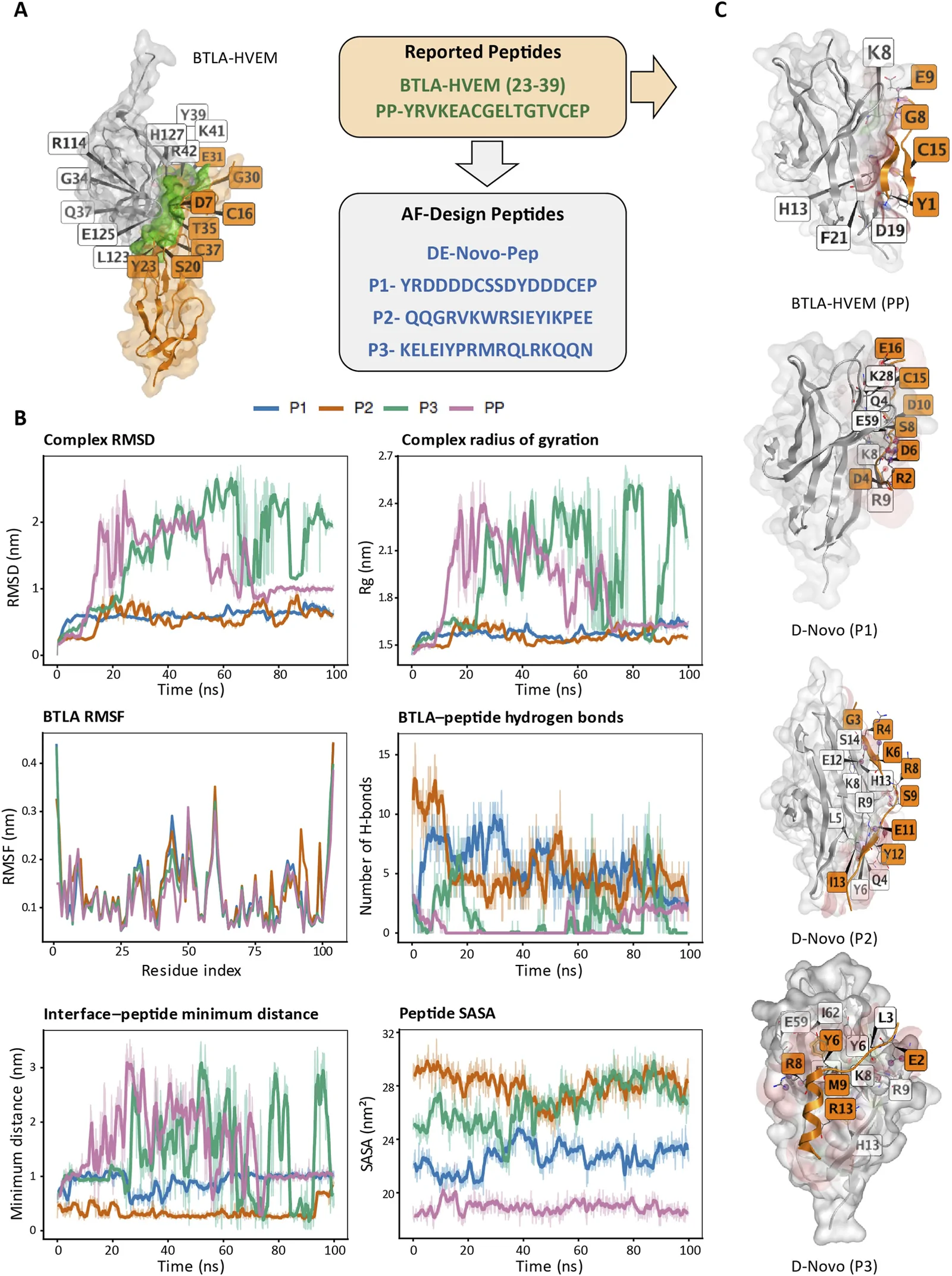
Structure guided design and molecular dynamics assessment of BTLA binding peptides. **(A)** Schematic overview of the BTLA-HVEM interface used for peptide design, showing the reported HVEM derived reference peptide and three *de novo* generated peptides. **(B)** Docked binding poses of BTLA-HVEM, D-Novo-P1, D-Novo-P2, and D-Novo-P3 on the BTLA surface, with interacting residues indicated. **(C)** Molecular dynamics simulation analyses of BTLA peptide complexes over 100 ns, including complex RMSD, radius of gyration, BTLA RMSF, hydrogen bond dynamics, peptide interface minimum distance, and peptide solvent-accessible surface area.

The *BTLA-HVEM* crystal structure was used to define the peptide design site on the *HVEM* contacting surface of *BTLA* (residues 35-43 and 118-128), which interact with the N-terminal *CRD1* region of *HVEM* reference (van Veen et al., 2017). Guided by this interface, ColabDesign/AFDesign generated three *de novo* 17 residue *BTLA* targeting peptides (P1-P3), and the *HVEM* derived sequence *YRVKEACGELTGTVCEP (HVEM 23–39)* was included as a reference peptide (Figure 6A). Docking analysis showed that the designed peptides bound to the canonical HVEM binding surface of BTLA, with predicted poses overlapping the experimentally defined interface. Among the candidates, P2 displayed the most favorable binding profile, adopting a compact orientation with broader contact coverage across key BTLA surface residues than P1, P3, or the reference peptide. Consistently, the BTLA-P2 complex achieved the best docking performance, with the most favorable HADDOCK score of −105.3 ± 4.9 and the lowest Z-score of −1.7, suggesting stronger and more reliable binding (Figure 6B; Supplementary Tables S4, S5).

Consistent with the docking results, 100 ns molecular dynamics simulations identified P2 as the most stable and interface retentive candidate. After equilibration, the *BTLA-P2* complex maintained a low and stable RMSD and a consistently compact radius of gyration R_g, indicating limited structural drift, and preserved the shortest peptide interface distance for most of the trajectory, during simulation. P2 also, supporting sustained engagement with the *BTLA* binding surface. In contrast, P3 and the reference peptide showed greater conformational fluctuation and intermittent displacement from the interface. Hydrogen bond analysis further supported P2 stability, as it formed abundant early contacts with *BTLA* and retained detectable interactions throughout the simulation. Comparable BTLA RMSF profiles across the complexes indicated that peptide binding did not evidently perturb receptor flexibility. Overall, the integrated docking and MD analyses support P2 as the lead *BTLA* binding peptide for downstream validation (Figure 6C).

## Discussion

Our integrative single cell RNA-sequencing (scRNA-seq) analysis, unlike prior studies focusing on primary breast tumors (Shi et al., 2022) or lymph node metastases alone (Xu et al., 2021), elucidates compartment specific immune features and conserved transcriptional signatures across anatomical sites (Azizi et al., 2018). This high resolution approach reveals substantial immune heterogeneity and dynamic functional reprogramming throughout the metastatic cascade. Tumors with increasing metastatic potential exhibit pronounced immune dysregulation, characterized by altered cytokine signaling and impaired effector responses, which correlate with poorer clinical outcomes (Xu et al., 2021). Chemokines and cytokines, including *CCL4*, *CCL5*, CXCR3, *CXCR4*, *CXCL13*, *IL2RG*, and *IL32*, emerge as pivotal modulators of immune infiltration, cellular differentiation, and phenotypic transitions, shaping the immunological architecture of the tumor microenvironment (TME) and driving metastatic progression (Winkler et al., 2024; Reeves, 2024). These observations align with prior evidence that metastatic breast tumors suppress anti-tumor immune signaling while promoting pathways that facilitate dissemination and immune escape (Davis et al., 2020).

T cells display significant functional variability across compartments. In tumors, CD8^+^ T cells are enriched for cytotoxic and exhausted phenotypes, while CD4^+^ subsets comprise both effector memory and regulatory T cells (Tregs), indicating an active yet dysregulated immune response (Núñez et al., 2020). Conversely, tumor draining lymph nodes (TLNs) harbor higher proportions of naïve and activated CD4^+^ T cells and immature B cells, consistent with a suppressive immunological milieu. Notably, T cell transcriptional profiles in TLNs closely resemble those in peripheral blood mononuclear cells (PBMCs), suggesting systemic immunological feedback and fluid immune cell trafficking between tumors and lymphoid tissues (Shi et al., 2022; Núñez et al., 2020). Both CD4^+^ and CD8^+^ T cells contribute to anti-tumor immunity but are frequently suppressed by regulatory subsets, particularly Tregs and regulatory B cells, within tumors and TLNs (Núñez et al., 2020). Differential expression of chemokines and cytokines, such as *CCL5*, which facilitates leukocyte trafficking and extracellular matrix remodeling, and *CXCL13*, which recruits B cells and T follicular helper cells to tertiary lymphoid structures, fosters immunosuppressive niches (Yi et al., 2024). Elevated IL-32 expression across compartments further supports inflammatory rewiring of the tumor stroma.

Following *PD-1* blockade, we observe compartment specific immune reprogramming. In tumors, transcriptional signatures enriched in interferon-gamma (IFN-γ) and interleukin signaling pathways indicate heightened immune sensitivity. In contrast, B cells in TLNs and PBMCs favor signaling programs linked to *VEGFA* and *TGF-β*, suggesting roles in immune-regulation and angiogenesis. Post-treatment, cytotoxic CD8^+^ and regulatory CD4^+^ T cells predominate, accompanied by a reduction in B cell subsets (Yi et al., 2024; Wang et al., 2023). Transcription factor (TF) network analysis reveals a shift in regulatory dominance post-therapy. Pre-treatment immune cells exhibit strong activation of *E2F1, RELA, GATA3*, and *ELF4*, indicative of proliferation and effector activation (Mamonkin et al., 2014; Qin et al., 2025). Post-therapy profiles show enriched activity of *FOXO1, IKZF1, NR3C1, and CREB3L2,* signaling a transition toward memory formation and lineage stabilization, reflecting adaptive reprogramming of lymphocytes for sustained immune surveillance (Ribas et al., 2016).

We further delineate the role of alternative immune checkpoint pathways. The *BTLA TNFRSF14* (*HVEM*) axis is prominently active in PBMCs and TLNs. *BTLA*, expressed on T cells, engages *HVEM* on B cells and antigen-presenting cells to suppress *IFN-γ* and promote apoptosis, contrasting with co-stimulatory signals mediated by *LIGHT* and *LTα*, which activate *NF-κB* signaling via distinct *HVEM* domains. This functional competition modulates immune tone and may contribute to resistance against *PD-L1* blockade (Zhang et al., 2025). The *CD74 CXCR4 CD44* signaling axis is considerably elevated in PBMCs and TLNs. *MIF* binding to *CD74* initiates proteolytic signaling that promotes cell survival and immune escape, while *CXCR4* and its ligand *CXCL12*, upregulated in the TME and post-treatment PBMCs, support therapy resistance and metastasis, particularly under hypoxic or DNA damage conditions. However, *CXCR4*’s broad expression across immune subsets poses challenges for systemic inhibition (Ning et al., 2021).

Cell-cell communication analyses highlight specific LR interactions central to immune dysregulation. The *CD70*-*CXCR4* axis exhibits high signal strength despite low interaction frequency in pre-treatment TLN samples (Sordo-Bahamonde et al., 2023; Becker-Herman et al., 2005). Post-treatment, this axis becomes broadly active across B and T cell subtypes but with reduced signaling intensity. *MIF-CD74*-*CXCR4* interactions are enriched in TLNs during early disease stages, particularly when B cells are the source, but diminish in later stages and tumors, suggesting early immune coordination via B-cell derived cues followed by immunosuppressive rewiring during progression. T-cell derived MIF interactions increase modestly in late stage TLNs, indicating potential adaptive feedback mechanisms (Wang et al., 2023). Checkpoint expression profiling reveals limited involvement of classical *PD-L1*/*PD-L2* pathways, with immune resistance dominated by non-canonical checkpoints like *BTLA* and *MIF*. This non-classical resistance signature underscores the limitations of *PD-1* monotherapy in certain breast cancer subtypes and highlights the need for combinatorial or alternative immunomodulatory strategies (Yi et al., 2024; Gore et al., 2008).

Post-therapy immune profiling shows a predominance of cytotoxic CD8^+^ and regulatory CD4^+^ T cells across compartments, with reduced B-cell representation (Bassez et al., 2021; Koh and Ellisen, 2021). Regulatory network analysis confirms a pre-treatment dominance of *E2F1, RELA, GATA3,* and *ELF4*, reflecting proliferative activation and effector differentiation. Following *PD-L1* blockade, enriched activity of *IKZF1, FOXO1, JUND, NR3C1,* and *CREB3L2* indicates a shift toward memory cell maintenance and B-T cell coordination, suggesting a reprogramming of lymphocytes from reactive to sustained immune effectors (Ribas et al., 2016; Zhang et al., 2025). Collectively, our findings position the *BTLA-HVEM* and *CD74*-*CXCR4* axes as central nodes in B-T cell crosstalk, contributing to immune escape and checkpoint inhibitor resistance. Disrupting these suppressive circuits may enhance immunotherapy efficacy, particularly in patients with immune-excluded or therapy refractory tumors.

The higher proportion of B and T lymphocytes in TLNs than in primary tumors is consistent with the role of tumor draining lymph nodes as sites of antigen drainage, dendritic cell mediated priming, and adaptive immune coordination (Delclaux et al., 2024; Pittet et al., 2023). By contrast, primary breast tumors often contain dominant malignant and stromal compartments, and immune excluded architecture may restrict lymphocyte entry into tumor nests (Almawash, 2025). In our data, TLN enrichment of adaptive immune cells may therefore reflect an active immune priming niche, with preserved naïve and memory T cell pools, B cell activation, and B-T cell communication. However, lymphocyte abundance alone does not imply effective antitumor immunity, because tumor draining or metastatic lymph nodes can also acquire dysfunctional, regulatory, or immunosuppressive programs (Delclaux et al., 2024). This distinction is important in the context of our observed BTLA-HVEM/TNFRSF14 signaling, which was enriched in TLNs and directed from B cells to T cells. Since BTLA-HVEM signaling can restrain T cell activation and regulate Tfh and B cell interactions, these findings suggest that TLN lymphocytes may be abundant but functionally constrained by checkpoint-dependent communication (Derré et al., 2010; Stienne et al., 2022). Consistent with this interpretation, our *de novo* HVEM mimetic peptide design provides an initial structure guided strategy to target the BTLA interface and potentially disrupt BTLA-HVEM mediated inhibitory signaling, although experimental validation will be required to confirm therapeutic activity (Pelay-Gimeno et al., 2015).

Despite these insights, our study is limited by its focus on immune cell transcriptomics, excluding tumor cell interactions that may critically influence immune remodeling. Additionally, the lack of experimental validation for inferred crosstalk mechanisms necessitates future mechanistic studies. High throughput screening of small-molecule compounds was not conducted in the present study. Such investigations represent a valuable avenue for future research, particularly following the successful validation of the peptide inhibitor as a proof-of-concept strategy for modulating this signaling pathway. Nevertheless, high resolution single cell profiling enables a comprehensive capture of complex immunological states and intercellular interactions across spatial and treatment contexts, laying a foundation for identifying novel therapeutic targets in metastatic breast cancer.

## Conclusion

Our single cell meta-analysis elucidates profound functional and phenotypic heterogeneity in B and T lymphocytes across breast cancer compartments, demonstrating that their crosstalk dynamically shapes the tumor immune microenvironment. Tumor-draining lymph nodes exhibit a distinct, compartmentalized program of T-cell mediated immunosuppression, alongside enrichment of plasmablasts and cytotoxic CD8^+^ T cells, consistent with B-T cell driven immune conditioning. Chemokine-cytokine circuits and non-classical immune checkpoints, notably the *MIF*-*CD74*-*CXCR4* axis and the *BTLA*-*TNFRSF14* (HVEM) pathway, emerge as pivotal regulators capable of shifting immune responses toward tumor promotion or control. In *BTLA-HVEM* context, therapeutic peptides may offer a promising approach to selectively modulate these signaling networks, providing a potential avenue for targeted intervention against these immune regulatory pathways. These findings enhance our understanding of lymphatic metastasis and position B- and T-cell associated signaling hubs as actionable biomarkers and potential targets for combination therapies to augment immunotherapy efficacy beyond *PD-1*/*PD-L1* blockade.

## Data Availability

All raw data analyzed in this study are publicly available from The Cancer Genome Atlas (TCGA) via the GDC portal and from the Gene Expression Omnibus (GEO; https://www.ncbi.nlm.nih.gov/geo/). Dataset accession numbers and processing details are provided in Supplementary Table S1.
