# Supplementary material for: Single cell mapping of B and T cell dynamics in breast cancer lymph node metastasis
Source: Front Bioinform. 2026 Aug 12;6:1833416. doi: 10.3389/fbinf.2026.1833416 (PMC13506802; doi:10.3389/fbinf.2026.1833416)
Supplement: Supplementary file 1 [file Supplementaryfile1.docx]

**Single Cell Mapping of B and T Cell Dynamics in Breast Cancer Lymph Node Metastasis**

**Sajid Khan, Sabhat Jamil, Muhammad Hamza, Zarlish Attique, and Suping Zhang**

**SUPPLEMENTARY DATA**

**Fig S1. UMAP visualization and cell-type distribution across breast cancer immune compartments from four single-cell RNA-seq datasets. (A-D)** UMAP plots illustrate immune cell clustering across distinct tissue compartments (normal, TLN/TdLN, and PBMC) in four publicly available datasets: GSE161529 **(A)**, GSE195861 **(B)**, GSE180286 **(C)**, and GSE263995 **(D)**. Immune cell populations were annotated based on canonical markers into B cells (red), T cells (purple), NK cells (blue), myeloid cells (green), and dendritic cells (teal, where applicable). Corresponding bar plots (right panels) display the proportion of each cell type across tissue types within each dataset. A consistent pattern of immune remodeling is observed, with tumors showing enrichment of T cells and NK cells, and TLNs exhibiting higher B cell representation. PBMCs retain a more balanced immune profile, reflecting systemic immune states.

**Fig S2. GSE167036 immune population analysis focusing on B and T cell compartments**. **(A-B)** Top: UMAP projections showing major immune and stromal cell types (left) and their corresponding sub-cell type annotations (right), with clusters labelled across the shared embedding. Bottom: same UMAP space split by sample origin, showing Lymph Node and Tumor cells (left), and then separated views highlighting cell type distribution within each sample group (right). **(C)** Stacked bar plots summarizing relative cell proportions across Lymph Node and Tumor samples at two levels: major cell types (left) and finer sub-cell types with emphasis on B and T cell compartments (right).

**Fig** **S3**. **Differential Pathway Enrichment and Functional Signatures in B and T Cells Across Breast Cancer Compartments. (A)** Bar plot showing comparative pathway enrichment analysis of B cells (green) and T cells (blue) based on transcriptomic data. Enrichment was determined using –log₁₀(q-value), highlighting distinct functional programming in each lymphocyte subset. T cells predominantly enriched pathways involved in cytotoxicity, antigen presentation, and checkpoint response (e.g., PD-1 blockade, IL-7 signaling), whereas B cells displayed upregulation of pathways related to cytokine signaling, immune regulation, and VEGFA/TGF-β-mediated remodeling. **(B)** Dot plot representing KEGG pathway enrichment in B and T cells across tumor-draining lymph nodes (TdLN/TLN) and tumor tissues. Dot color reflects statistical significance (–log₁₀(q-value)) and size indicates gene count involved in each pathway. B cells in tumors showed elevated oxidative phosphorylation and endoplasmic reticulum stress pathways, while T cells exhibited antigen presentation, Toll-like receptor signaling, and TNF signaling pathway enrichment. Distinct pathway usage between TdLN and tumor highlights tissue specific immune programming and functional specialization.

**Fig S4. Distinct ligand-receptor communication patterns shaping B and T cell immunological states across breast cancer compartments.** Sankey plots illustrate incoming (top) and outgoing (bottom) cell-cell communication patterns inferred from ligand-receptor interactions among B and T cell subsets. Each cell group (left column) is mapped to a distinct communication pattern (middle columns), which in turn links to key signaling pathways (right column) such as MK, SPP1, CXCL, IL16, GDNF, VISFATIN, and GRN. The width of the connections represents the contribution strength of each cell group or signaling axis to the overall communication pattern. Notably, incoming signals in Pattern 1 were predominantly directed toward B_Plasma and B_Memory cells, while outgoing communication in Pattern 3 originated mainly from CD4_Tfh and CD8_Cytotoxic subsets. These communication routes reflect dynamic regulatory interactions shaping immune behavior across the metastatic cascade.

**Fig S5. Cell cell communication analysis between immune cell populations in tumor and lymph node samples (GSE167036). (A)** Bar plots showing overall outgoing and incoming communication strength for major B and T cell compartments, with lymph node and tumor shown side by side tumor (red) and lymph node (cyan green). Lower plots show the same comparison at the signaling program level, ranking the main outgoing and incoming interaction features across conditions. **(B)** Dot plots summarizing selected ligand receptor interactions, including MIF associated signaling, across B cell and T cell sender receiver pairs. Dot color represents communication probability or average expression, while dot size indicates interaction probability or significance as shown in each legend. **(C)** Network diagrams showing inferred communication among CD4+ T cells, CD8+ T cells, B cells, and plasma B cells in tumor and lymph node samples. Line thickness reflects relative interaction strength, and edge labels indicate communication weights between cell populations.

**Fig S6. Comparative enrichment of hallmark cancer pathways in immune cells pre- post-treatment.** Radar plots illustrate the adjusted p-values of hallmark cancer pathways enriched in immune cell transcriptomes before (top) and after (bottom) treatment. Each spoke represents a canonical cancer hallmark (e.g., immune evasion, angiogenesis, apoptosis resistance), and the bar length indicates statistical significance (adjusted p-value). Pre-treatment immune cells prominently exhibited signatures related to immune suppression, inflammation, and resistance to cell death, whereas post-treatment samples showed reduced enrichment in these programs and a shift toward pathways associated with immune activation, angiogenesis, and tumor invasion. These results underscore therapy-induced reprogramming of the tumor immune landscape.

**Fig S7. Differential ligand-receptor communication between B and T cells across tissues, conditions, and directions in breast cancer.** Chord diagrams display inferred cell-cell interactions between B and T cell populations across tissues (Tumor, TdLN, PBMC) and treatment conditions (Pre vs. Post), with directional comparisons (B→T and T→B). Each arc represents a ligand-receptor pair, where the thickness denotes interaction strength, and color denotes the cell type of origin. Notably, TdLN samples exhibited a broader spectrum and strength of interactions pre-treatment (particularly in B→T direction), while post-treatment samples displayed increased diversity but reduced signal intensity. Tumor samples revealed diminished communication complexity, suggesting an immunosuppressive microenvironment. The directional asymmetry highlights context-dependent rewiring of immune crosstalk, implicating tissue- and treatment-specific immunomodulatory landscapes.

**Supplementary Table S1.** National Center for Biotechnology Information (NCBI) Gene Expression Omnibus (GEO) datasets used for profiling tumor and lymph node metastases in breast cancer.

| Data Set | Total Patients / Samples | Patient data use in this study | Tumor samples | Lymph node metastasis samples | B Cells | T Cells | Platform | PMCID | |
| --- | --- | --- | --- | --- | --- | --- | --- | --- | --- |
| GSE161529 | 52 patients | 6 ER+ lymph-node metastases Patient | 20 ER+ Tumor | 6 ER+ lymph-node | 3021 | 5177 | GPL18573 Illumina NextSeq 500 | PMC10259667 | |
|  | 69 samples | 26 samples |  |  |  |  |  | PMC10933444 | |
| GSE195861 | 14 patients | 14 patients | 14 Tumor (8 DCIS, 6 IDC) | 6 lymph node metastasis samples (paired with all IDC cases) | 11411 | 7058 | GPL20795 HiSeq X Ten | PMC11448450 | |
|  | 20 samples | 20 samples |  |  |  |  |  |  |  |
|  |  |  |  |  |  |  |  |  |  |
| GSE180286 | 5 patients | 5 breast cancer | 5 Tumour | 10 lymph node | 30955 | 27460 | GPL20795 HiSeq X Ten | PMC10576881 | |
|  | 15 samples | 15 samples |  |  |  |  |  |  |  |
| GSE263995* | 2 TNBC patients | 2 TNBC patients | 6 Tumour (3 Pre_Post) | 4 lymph node (2 TdLN Pre_Post), and 2 (PBMC Pre_Post) | 23740 | 43665 | GPL24676 Illumina NovaSeq 6000 | PMC11134561 | |
|  | 14 samples | 14 samples |  |  |  |  |  |  |  |
| Combined |  | 27 patients | 45 Tumor | 26 lymph node metastasis | **59101** | **83884** |  | | |
| Test Data Set GSE167036 | 32 | 32 | 16 Tumor | 16 matched lymph node metastasis (LNMT) | **24998** | **54736** | GPL24676  Illumina  NovaSeq 6000 | | [PMC9649678](https://pmc.ncbi.nlm.nih.gov/articles/PMC9649678/) |

*GSE263995 patient receive chemo therapy

**Supplementary Table S2.** List of signature genes used for major cluster identification and subtype annotation in scType analysis (*Reference*: https://doi.org/10.1002/cac2.12429).

| **Cluster** | **Canonical genes** | **Signature genes** |
| --- | --- | --- |
| CD4_Naive | CD3D, CD3E, CD3G, CD4, CD40LG | CCR7, SELL, EEF1G, LDHB, MYC |
| CD4_Activated |  | CCL20, CD40LG, CD69, JUNB, FOS |
| CD4_Tcm |  | LMNA, ANXA1, CCR7, ANXA2, ITGB1 |
| CD4_Tem |  | KLRB1, AQP3, CCR6, BHLHE40, LTB |
| CD4_Tfh |  | CXCL13, CTLA4, BATF, PDCD1, ICOS |
| CD4_Treg |  | FOXP3, IKZF2, CTLA4, IL2RA, TIGIT |
| CD8_Naive | CD3D, CD3E, CD3G, CD8A, CD8B | YBX3, CD8A, TGFB1, CCL5, CXCR3 |
| CD8_Activated |  | HSPA1B, FOSB, DUSP1, FOS, CD69 |
| CD8_Cytotoxic |  | GZMB, NKG7, CST7, GZMK, PRF1 |
| CD8_Exhausted |  | CXCL13, HAVCR2, LAG3, PDCD1, CTLA4 |
| CD8_Trm |  | KLRC1, KLRD1, CD69, IFNG, NKG7 |
| B_Naive | CD79A, CD79B, MS4A1, CD19 | YBX3, IGHD, IL4R, FCER2, SELL |
| B_Memory |  | CRIP1, S100A10, VIM, ANXA2, HSPA1B, DNAJB1, HSPA1A, DNAJA1, NR4A2,ZBTB32, MYC, IL2RA, IRF4 |
| B_Plasma |  | IGHG1, IGHG4, IGHG3, JCHAIN, MZB1, XBP1, PRDM1, CD38 |
| B_PB |  | MKI67, UBE2C, TOP2A, CD38, IGHG4, MZB1, PRDM1 |
| NK_Naive | GNLY, NKG7, TYROBP, PRF1 | IL7R, TCF7, XCL1, CCR7, SELL |
| NK_Activate |  | HSPA1B, CCL3, IFNG, TNF, CD69 |
| NK_Cytotoxic |  | ZNF683, TYROBP, GZMA, GNLY, NKG7 |
| NK_Exhausted |  | PRF1, HAVCR2, TNFRSF1B, KLRF1, KLRB1 |
| NKT_Activated |  | GZMA, CCL4, IFNG, CD69, KLRG1, CD3E |
| NKT_Exhausted |  | YBX3, ANXA2, ANXA5, CD3D, ZBTB7A, IL7R |
| M_Macro | CST3, LYZ | APOE, C1QB, C1QA, CD9, CD276, SPP1 |
| M_Macro |  | IL1B, S100A9, VCAN, FCN1, S100A12 |
| M_Mono |  | FCN1, LYZ, CD163, CLEC12A, CD14 |
| M_cDC |  | CLEC10A, FCER1A, CD1C, CST3, CCR7,CST3, CRIP1, CLEC9A, S100A10, IDO1, LYZ |
| M_pDC |  |  |
|  |  | JCHAIN, IRF4, IGHG4, TCL1A, CLEC4C |
| M_Neutro |  | CXCL8, CXCL1, S100A8, S100A9, CSF3R |
| M_Mast |  | TPSB2, CPA3, GATA2, MS4A2, FCER1A |

**Supplementary Table S3.** Condition-specific cell-type composition and ANOVA.

| **Cell Types** | **Baseline**  **Prop** | **PropMean**  **PBMC** | **PropMean**  **TLN** | **PropMean**  **Tumor** | **Fstatistic** | **P.Value** | **FDR** |
| --- | --- | --- | --- | --- | --- | --- | --- |
| M | 0.1412280 | 0.14785199 | 0.19541206 | 0.55328227 | 17.290960 | 5.441520e-07 | 2.176608e-06 |
| B | 0.2987146 | 0.03440411 | 0.35159433 | 0.13269450 | 14.688080 | 3.538608e-06 | 7.077215e-06 |
| NK | 0.1332782 | 0.57821238 | 0.08741005 | 0.08494279 | 11.111428 | 5.376246e-05 | 7.168329e-05 |
| T | 0.4267792 | 0.23953151 | 0.36558356 | 0.22908044 | 3.741371 | 2.787018e-02 | 2.787018e-02 |

**Supplementary Table S4.** Comparative hydrogen-bond interaction profile of BTLA-peptide complexes.

| **Complex** | **Total H-bonds** | **Energy minimum** | **Energy maximum** | **Mean energy** | **Distance minimum** | **Distance maximum** |
| --- | --- | --- | --- | --- | --- | --- |
| **BTLA-PP** | 4 | -2.90 | -0.50 | -1.23 | 3.41 | 4.39 |
| **BTLA-P1** | 11 | -8.90 | -0.70 | -3.45 | 2.80 | 3.52 |
| **BTLA-P2** | 11 | -8.90 | -0.70 | -3.45 | 2.80 | 3.52 |
| **BTLA-P3** | 6 | -8.00 | -0.50 | -3.08 | 2.85 | 3.98 |

**Supplementary Table S5.** Comparison of docking scores and interface characteristics of BTLA-peptide complexes.

| **Parameters** | **BTLA-PP** | **BTLA-P1** | **BTLA-P2** | **BTLA-P3** |
| --- | --- | --- | --- | --- |
| HADDOCK score | -95.2 +/- 4.6 | -56.4 +/- 4.3 | -105.3 +/- 4.9 | -55.5 +/- 5.2 |
| Cluster size | 13 | 14 | 15 | 12 |
| RMSD from the overall lowest-energy structure | 0.3 +/- 0.2 | 10.2 +/- 0.1 | 0.5 +/- 0.3 | 10.3 +/- 0.2 |
| Van der Waals energy | -59.1 +/- 4.2 | -32.7 +/- 3.0 | -48.5 +/- 6.3 | -28.7 +/- 4.3 |
| Electrostatic energy | -277.3 +/- 10.7 | -179.6 +/- 28.3 | -320.9 +/- 19.9 | -212.2 +/- 13.9 |
| Desolvation energy | -4.7 +/- 0.8 | -2.9 +/- 1.9 | -13.1 +/- 2.0 | -0.2 +/- 2.2 |
| Restraints violation energy | 170.5 +/- 32.9 | 150.4 +/- 24.0 | 205.9 +/- 39.6 | 159.4 +/- 15.1 |
| Buried Surface Area | 1524.2 +/- 26.4 | 1328.9 +/- 33.1 | 1643.8 +/- 38.8 | 1293.2 +/- 28.5 |
| Z-Score | -1.6 | -1.4 | -1.7 | -1.4 |
